# Variation in the metagenomic analysis of fecal microbiome composition calls for a standardized operating approach

**DOI:** 10.1128/spectrum.01516-24

**Published:** 2024-10-30

**Authors:** Zhilu Xu, Yun Kit Yeoh, Hein M. Tun, Na Fei, Jingwan Zhang, Mark Morrison, Michael A. Kamm, Jun Yu, Francis Ka Leung Chan, Siew C. Ng

**Affiliations:** 1 Microbiota I-Center (MagIC), Hong Kong SAR, China; 2 Department of Medicine and Therapeutics, The Chinese University of Hong Kong, Hong Kong SAR, China; 3 Li Ka Shing Institute of Health Sciences, State Key Laboratory of Digestive Disease, Institute of Digestive Disease, The Chinese University of Hong Kong, Hong Kong SAR, China; 4 Australian Institute of Marine Science, AIMS@JCU, Townsville, Queensland, Australia; 5 JC School of Public Health and Primary Care, The Chinese University of Hong Kong, Hong Kong SAR, China; 6 Department of Medicine, University of Chicago, Chicago, Illinois, USA; 7 The University of Queensland, Diamantina Institute, Faculty of Medicine, Brisbane, Australia; 8 Department of Gastroenterology, St Vincent’s Hospital, Melbourne, Australia; 9 Centre for Gut Microbiota Research, Faculty of Medicine, The Chinese University of Hong Kong, Hong Kong SAR, China; Lerner Research Institute, Cleveland, Ohio, USA

**Keywords:** microbiome, metagenomics, DNA extraction, batch effect

## Abstract

The reproducibility in microbiome studies is limited due to the lack of one gold-standard operating procedure. The aim of this study was to examine the impact of protocol variations on microbiome composition using metagenomic data sets from a single center. We assessed the variation in a data set consisted of 2,722 subjects, including 9 subcohorts harboring healthy subjects and patients with various disorders, such as inflammatory bowel disease, colorectal cancer, and type 2 diabetes. Two different DNA extraction kits, with or without lyticase, and two sample storage methods were compared. Our results indicated that DNA extraction had the largest impact on gut microbiota diversity among all host factors and sample operating procedures. Healthy subjects matched by age, body mass index, and sample operating methods exhibited reduced, yet significant differences (PERMANOVA, *P* < 0.05) in gut microbiota composition across studies. The variations contributed by DNA extraction were primarily driven by different recovery efficiency of gram-positive bacteria, e.g., phyla Firmicutes and Actinobacteria. This was further confirmed by a parallel comparison of fecal samples from five healthy subjects and a standard mock community. In addition, the DNA extraction method influenced DNA biomass, quality, and the detection of specific lineage-associated diseases. Sample operating approach and batch effects should be considered for cohorts with large sample size or longitudinal cohorts to ensure that source data were appropriately generated and analyzed. Comparison between samples processed with inconsistent methods should be dealt with caution. This study will promote the establishment of a sample operating standard to enhance our understanding of microbiome and translating in clinical practice.

**IMPORTANCE:**

The reproducibility of human gut microbiome studies has been suboptimal across cohorts and study design choices. One possible reason for the disagreement is the introduction of systemic biases due to differences in methodologies. In our study, we utilized microbial metagenomic data sets from 2,722 fecal samples generated from a single research center to examine the extent to which sample storage and DNA extraction influence the quantification of microbial composition and compared this variable with other sources of technical and biological variation. Our research highlights the impact of DNA extraction methods when analyzing microbiome data and suggests that the microbiome profile may be influenced by differences in the extraction efficiency of bacterial species. With metagenomics sequencing being increasingly used in clinical biology, our findings provide insight into the challenges using metagenomics sequencing in clinical diagnostics, where the detection of certain species and its abundance relative to a “healthy reference” is key.

## INTRODUCTION

The human gut microbiome is emerging as a crucial factor in human diseases development (1–4). Metagenomic sequencing provides increased species resolution and information on the functional potential of the gut microbiome (5). However, the reproducibility and predictive accuracy of these high-resolution microbial signatures have often been suboptimal when comparing across cohorts (6). One possible reason for the discrepancy is the introduction of systemic biases due to differences in study design, including methodologies and analytical workflows across studies (6, 7). These biases begin with sample collection and continue to be introduced throughout the entire experiment leading to an observed community that is significantly altered from the true underlying microbial composition (8). Identifying how factors in sample operation approach contribute differently to these microbial biases between studies is essential and requires the use of highly sensitive metagenomic sequencing techniques in a large cohort under different clinical conditions.

Sample operating steps have a remarkable influence on investigating the gut microbiome due to their impact on DNA extraction yield, richness, microbial composition, and the recovery of specific bacterial lineages. Great effort has been made to decipher the impact of each sample operating step, and DNA extraction has been shown to have the largest effect on metagenomic analysis outcomes compared to library preparation and sample storage (6, 9). However, methodology toward better DNA extraction has been under inconstant development over time, and each protocol has its limitations (6, 10). For instance, the well-known Qiagen company launched the PowerFecal Pro Kit for fecal DNA extraction to substitute the previous legacy kits for fecal DNA extraction. While the PowerFecal Pro Kit was equipped with an upgraded version of Inhibitor Removal Technology and yields improvement in DNA quality, the resulting microbiome composition also varied from previous kits (11). As a consequence of the emerging new commercial solutions, it is sometimes inevitable for large research groups to adjust sequencing protocols over time and its dilemma whether to switch to more updated DNA extraction kits and protocols for large prospective cohorts. Thus, it is crucial to evaluate the influence of these DNA extraction kits on microbial profile comparison across studies recruited at different times, even though in the same center.

In our study, we utilized microbial metagenomic data sets from 2,722 fecal samples generated from an ethnically homogenous population to examine the extent to which sample storage and DNA extraction influence the quantification of microbial composition. In addition to the real data sets, we also included a mock community with predefined microbial composition, including three gram-negative and five gram-positive bacterial species and two fungal species. We aimed to assess the contributions of sample operating protocols on microbial profile bias through various DNA quality and quantity, microbial diversity, and composition, and address the challenges of combining sequenced data sets from cohorts collected at different timepoints to avoid errors for statistical analysis and biological conclusions.

## MATERIALS AND METHODS

### Study participants

The current data set included metagenomic sequencing data from 2,722 subjects including disease-free individuals and patients diagnosed with various diseases, such as inflammatory bowel disease, colorectal cancer (CRC), type 2 diabetes, coronavirus disease 2019 (COVID-19), and autism spectrum disorder (ASD). Among them, 881 were collected from Yunnan by the First Affiliated Hospital of Kunming Medical School. The study was approved by the Institutional Review Board and Research Ethics Committee of the First Affiliated Hospital of Kunming Medical School (Ref. No: 2017.L.14). Remaining subjects were recruited during their medical visit in two hospitals and a health center (Prince of Wales Hospital, United Christian Hospital, and tertiary referral center) in Hong Kong for various studies. All studies were approved by the Joint Chinese University of Hong Kong, New Territories East Cluster Clinical Research Ethics Committee. Written informed consents were obtained from all subjects.

All participants were recruited and diagnosed from January 2017 to March 2022. Subjects with CRC and carcinoma were diagnosed by colonoscopy and confirmed on histology examinations; subjects with Crohn’s disease (CD) were diagnosed based on standard criteria of endoscopy, radiology, and histological examinations. Obesity was defined as subjects with a body mass index (BMI) of over 28. All subjects apart from the obesity group had a normal range of BMI of 18.5–22.9. Patients were excluded if they had the following: age under 18 or over 80; self-reported comorbidities of other diseases; infection with an enteric pathogen; acquired immunodeficiency syndrome; known history of organ dysfunction or failure and abdominal surgery; active malignancy or undergoing radio-chemotherapy; short bowel syndrome; taking drugs commonly known to affect the gut microbiome, including proton pump inhibitors, oral non-steroidal anti-inflammatory drugs, corticosteroids, laxatives, or selective serotonin reactive inhibitors and antibiotics or probiotics use within 3 months of sample collection; pregnant or breastfeeding; on special diets such as vegetarians.

Healthy controls were recruited during the same recruitment period from the community through advertisement and from the endoscopy center at the Prince of Wales Hospital and included subjects who had a normal colonoscopy (fecal samples collected before bowel preparation). The exclusion criteria for healthy controls were known complex infections or sepsis; known history of severe organ failure (including decompensated cirrhosis, malignant disease, kidney failure, epilepsy, active serious infection, acquired immunodeficiency syndrome); bowel surgery in the last 6 months (excluding colonoscopy/procedure related to perianal disease); the presence of an ileostomy/stoma; and current pregnancy; any long-term drugs for chronic diseases; the use of antibiotics in the last 3 months; the use of laxatives or anti-diarrheal drugs in the last 3 months; or recent dietary changes (e.g., becoming vegetarian/vegan).

Fecal samples were collected from all participants. Stool samples were either freshly frozen or immersed in a preservative solution (Norgen Biotek, catalog No. 28330). Fecal specimens were stored at −80°C until further processing. Study characteristics are summarized in Table 1.

**TABLE 1:** Study characteristics^*a*^

Study ID^*b*^	Location	DNA extraction	Preservative	Lyticase	Disease	Sample size (disease)	Sample size (control)
S1 (12)	HK	Qiagen QIAamp DNA Stool Mini Kit	N	N	CRC	339	193
S2 (13)	HK	Qiagen DNeasy PowerSoil Kit	Y	N	NA	0	546
S3 (14)	HK	Promega PureFood	N	Y	ObT2	73	64
S4 (15)	HK	Promega PureFood	Y	Y	ASD	64	64
S5 (16)	Yunnan	Promega PureFood	N	Y	NA	0	881
S6	HK	Promega PureFood	N	Y	CRC	134	30
S7	HK	Promega PureFood	N	Y	CD	92	63
S8 (17)	HK	Promega PureFood	Y	N	COVID-19	101	78

^*a*^ NA, not applicable; ObT2, obesity and type 2 diabetes; N, no; Y, yes.

^*b*^ Studies were ordered according to sample collection time.

### DNA extraction

The major distinction in DNA extraction protocols across data sets included in this study was the inclusion of a lyticase pretreatment and the choice of DNA extraction kit (Table 1). We briefly describe the protocols below for ease of reference. Exact protocols are available from the respective studies. DNA quality (A260/A280) was measured using NanoDrop ND-1000 spectrophotometer. DNA quantity was measured using a NanoDrop or Qubit Flex Fluorometer. DNA was stored at −20°C until use.

#### DNA extraction with Maxwell RSC PureFood GMO and Authentication Kit

DNA was extracted from 100 mg homogenized fractions of stool using a Maxwell RSC PureFood GMO and Authentication Kit (Promega) following the manufacturer’s instructions. Briefly, 1 mL of cetyltrimethylammonium bromide buffer was added to the pellet and vortexed for 30 s, and then the solution was heated at 95°C for 5 min. Samples were then vortexed thoroughly with beads (Biospec, 0.5 and 0.1 mm, 1:1) at maximum speed for 15 min. Following this, 40 mL proteinase K and 20 mL RNase A were added and incubated at 70°C for 10 min. The supernatant was then obtained by centrifuging at 13,000 g for 5 min and placed in a Maxwell RSC instrument for DNA extraction.

#### DNA extraction with Qiagen Kits

DNA was extracted from 100 mg homogenized fractions of stool using the Qiagen DNeasy PowerSoil Kit (13) or the Qiagen QIAamp DNA Stool Mini Kit (18) according to manufacturer’s instructions as previously described.

#### Pretreatment with lyticase

Lyticase was applied to increase the efficacy of fungal DNA extraction in several studies. Approximately 100 mg from each stool sample was prewashed with 1 mL ddH_2_O and pelleted by centrifugation at 13,000 g for 1 min. The pellet was resuspended in 800 mL TE buffer (pH 7.5), supplemented with 1.6 mL 2-mercaptoethanol and 500 U lyticase (Sigma), and incubated at 37°C for 60 min. The sample was then centrifuged at 13,000 g for 2 min, and the supernatant was discarded.

### Metagenomic sequencing

Extracted DNA was subjected to DNA libraries construction, completed through end repairing, adding A to tails, purification, and PCR amplification using Nextera DNA Flex Library Preparation Kit (Illumina, San Diego, CA). Libraries were subsequently sequenced on an Illumina NovaSeq 6000 System (2 × 150 bp) at the Centre for Gut Microbiota Research, Chinese University of Hong Kong, or by Novogene, Beijing, China. Comparisons between the Qiagen PowerSoil Kit and Promega Maxwell PureFood Kit were made possible by including a standardized mock community sequencing control consisting of five gram-positive bacterial species (*Bacillus subtilis*, *Listeria monocytogenes*, *Staphylococcus aureus*, *Enterococcus faecalis*, *Lactobacillus fermentum*), three gram-negative bacterial species (*Salmonella*, *Escherichia coli*, *Pseudomonas aeruginosa*), and two fungal species (*Saccharomyces cerevisiae*, *Cryptococcus neoformans*) (ZymoBIOMICS Microbial Community, catalog number D6300, Zymo Research, Irvine, California). In addition, five fecal samples from five healthy subjects were included in the comparison between DNA extraction kits. Stool samples were fresh frozen and processed within 1 month. The mock community solution was treated as a fecal sample when performing DNA extraction along with real fecal samples.

### Bioinformatic analysis

Metagenomic reads were quality filtered and trimmed using Trimmomatic (19) (v0.38) and decontaminated against the human genome (reference: hg38) using Kneaddata (v0.7.2, https://github.com/biobakery/kneaddata). Total number of reads after quality filtering and removal of human reads was used to represent sequencing depth, which may impact the detection rate of certain microbial taxa (20). For bacteriome, MetaPhlAn3 (21) (v3.0.9) was used to generate species-level abundance estimates against mpa_v30_CHOCOPhlAn_201901 database. The resulting abundance tables were processed in R v3.6.0 and tidyverse (22) (v1.2.1), ggpubr (23) (v0.2) R packages. Phyloseq (v1.24.2) R package was used for principal coordinates analysis (PCoA) to assess dissimilarities between samples based on weighted UniFrac distance (24). Vegan package was used to perform PERMANOVA test, Shannon index, and Chao1 richness (25). Multiple comparisons were performed using the Kruskal-Wallis test (KW) and post hoc Mann-Whitney test with Bonferroni corrections. Normalization of microbial community composition between studies was done using MMUPHin R package. Spearman’s correlation analysis between overall alpha-diversity and diversity within each bacterial phylum was performed in R. Statistical significance was taken as *P* < 0.05.

## RESULTS

### Significant inter-study variations were found in the gut microbiome profile of clinical subjects

**Fig 1:**
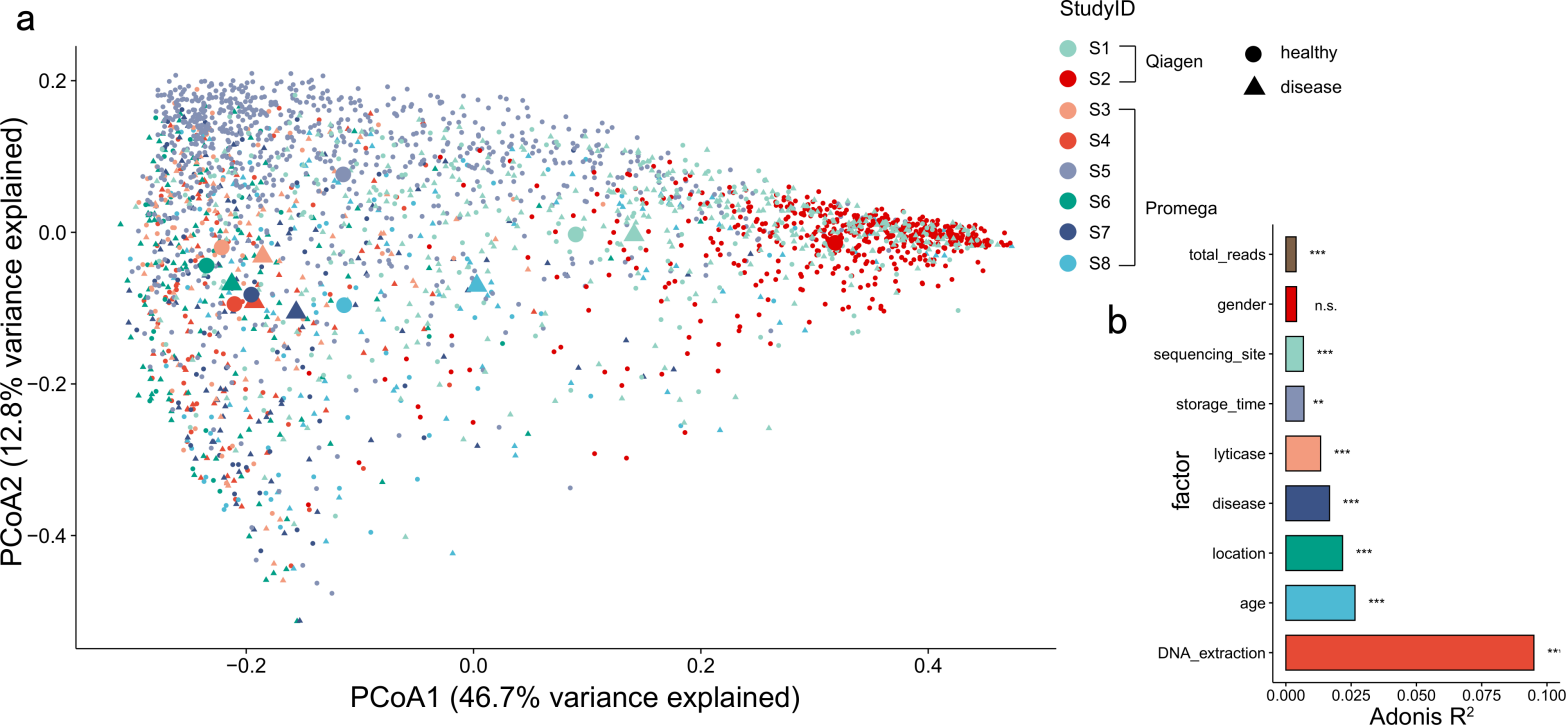
Significant inter-study variations were found in the microbiome profile of healthy subjects. (**a**) Principal coordinates plot based on weighted UniFrac distance showing variation among samples from individual studies. The variations were derived from between-sample weighted UniFrac distances. (**b**) Bar plot illustrating the variance explained (*R*^2^) by each factor associated with gut microbial variations. *R*^2^ and statistical significance were calculated by PERMANOVA (Adonis2). FDR was controlled at 5%. Factors were ranked by *R*^2^.

To evaluate the contributions of different factors to the microbial profile shift, we first compared the inter-study variations in the microbial profiles across different studies. In total, nine studies were included (S1 to S9) in the present research, with samples from studies S1 and S2 extracted with the Qiagen Kit and the others with the Promega Kit. At the whole bacteriome community level, fecal microbiomes primarily clustered according to study IDs irrespective of case-control groupings across all of the studies showed by weighted UniFrac distance analysis (Fig. 1a). Among these variables such as host factors (BMI, age, etc.) and sample preparation protocols, storage time and DNA extraction methods had the largest effect on gut microbiome composition (PERMANOVA test, *R*^2^ = 0.095, *P* < 0.001), followed by age, geography, health status, lyticase pretreatment, storage time, sequencing site, and sequencing depth (Fig. 1b). We then compared microbiome profiles between samples matched for age, BMI, sample storage conditions, and DNA extraction methods (40 pairs of matched samples from healthy subjects in studies S3, S7, DNA extracted by Promega Kit). We still observed significant differences in bacterial community composition between healthy subjects across individual studies (PERMANOVA test, *R*^2^ = 0.042, *P* = 0.005, Fig. S1a). When combined with the diseased subjects in the respective study, the variations contributed by the healthy status became dominant (*R*^2^ = 0.011, *P* = 0.011) compared to that by the study (*R*^2^ = 0.028, *P* = 0.001, Fig. S1b and c). These results indicated that bias between studies decreased after controlling the DNA extraction method and the sample storage condition. These results indicate that multiple factors determine the inter-study variations in the microbiome profile of clinical subjects, with sample operating approach, especially the DNA extraction method, contributed dominantly.

### Consistent shifts in microbiome composition between healthy subjects and patients were observed across studies

To find out if there were consistent shifts in microbiome, we then evaluated the microbial profile shift due to the healthy status across studies. Despite large inter-study variation in fecal microbiome profiles, subjects with various diseases showed shifts in the same direction along the PCoA1 axis compared with healthy controls in their respective study (Fig. 1a; Fig. S2), suggesting that the microbiome profiles from subjects with various diseases still share signatures compared with those from healthy subjects despite the inter-study variations. After adjusting for batch effect and multiple factors (age, BMI, sample storage, and DNA extraction methods), the effect size of inter-study variation reduced but still significant (PERMANOVA test, *R*^2^ = 0.02, *P* < 0.005, Fig. S3a and b). Patients still showed shifts in the same direction along the PCoA1 axis compared with healthy controls in their respective study (Fig. S3c). After adjustment, *Ruminococcus bromii*, *Faecalibacterium prausnitzii*, *Fusicatenibacter saccharivorans,* and *Bifidobacterium adolescentis* ranked top four species associated with the healthy state (FDR adjusted *P* < 0.05, Fig. S4). These observations indicated that consistent patterns in disease gut microbiota composition across studies were observed regardless of the bias between studies due to multiple factors.

### Inter-study variations were associated with variable detection sensitivity of certain bacterial groups

**Fig 2:**
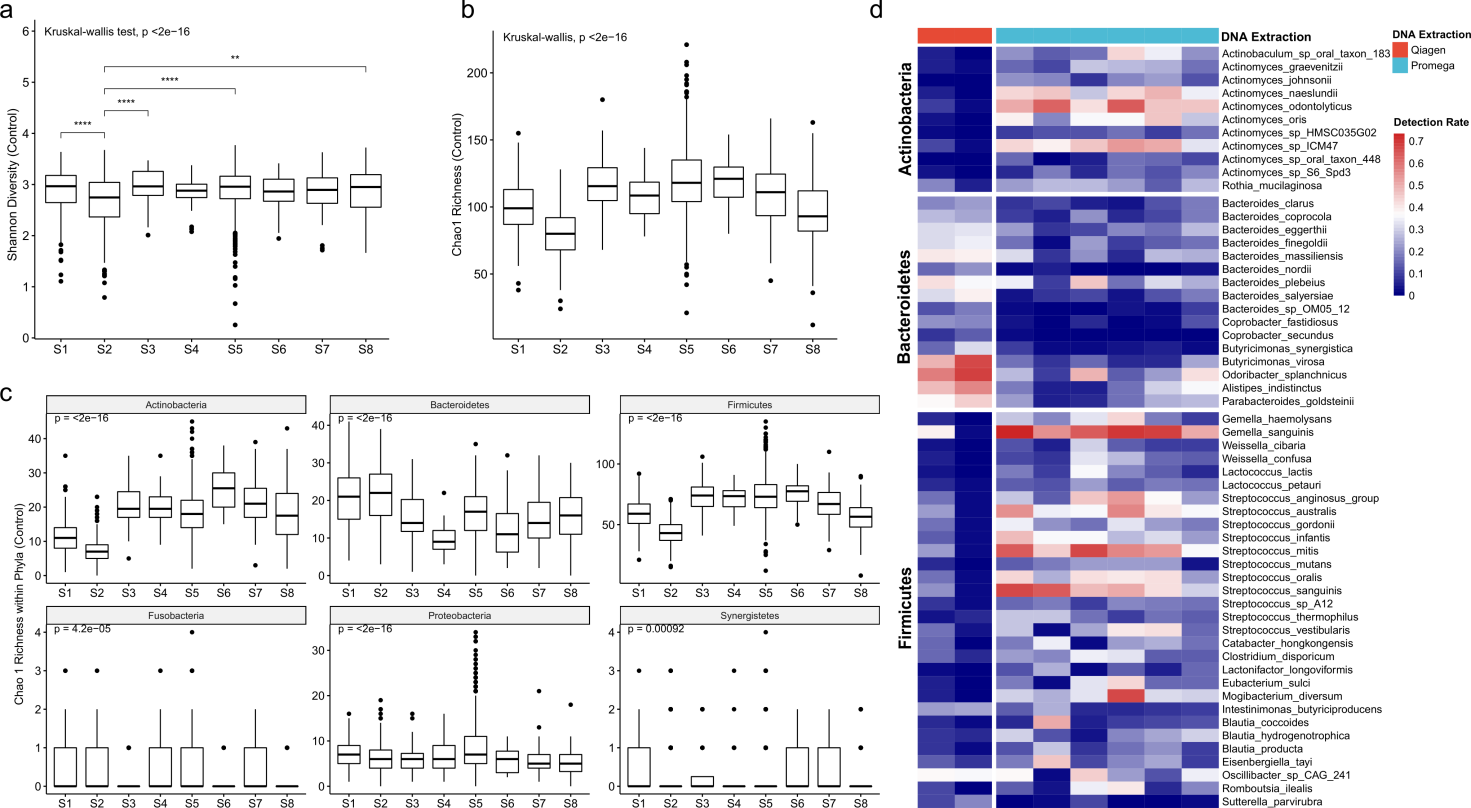
Microbial diversity and detection of bacterial groups. (**a**) Shannon diversity index of control samples across all included study. (**b**) Chao1 richness across all included study. (**c**) Bacterial richness within individual bacterial phyla across all included study. (**d**) Heatmap depicting the presence and absence of bacterial species in the major bacterial phyla across all included study. The results showed that the inter-study variations were largely caused by reduced detection of certain bacterial groups. Statistical significance was calculated by Kruskal-Wallis test.

To evaluate the factors that led to the inter-study variations, we next investigated the changes in microbiome alpha diversity and composition. When looking at data from healthy subjects, we found that the Shannon diversity index was relatively stable across most of control samples from different studies. The post hoc analysis with Bonferroni correction showed that only one study (S2) was significantly lower than the rest of the studies (*P* < 2e−16, Kruskal-Wallis test, Fig. 2a). However, the Chao1 richness showed large variations across individual studies (*P* < 2e−16, Fig. 2b). The Promega Kit-extracted samples exhibited significantly higher richness than the Qiagen Kit (*P* < 2e−16, Mann-Whitney test with Bonferroni correction). Notably, with the same DNA extraction method, the richness of samples stored in preservative was significantly lower than those of fresh-frozen samples [Qiagen, S1 (fresh frozen) vs S2 (preservative); Promega S3, 5–7 (fresh frozen) vs S4 and 8 (preservative), *P* < 0.05, Mann-Whitney test with Bonferroni correction]. At higher taxonomic rank, the dominant phyla observed in studies S1 and S2 (Qiagen) were Firmicutes [24.7% (16.1–37.4)%, median (P25-P75)] and Bacteroidetes [68.3% (48.4–79.1), median (P25-P75)], while the dominant phyla in studies S3–S8 (Promega) were Firmicutes [71.2% (56.6–82.9), median (P25-P75)] and Actinobacteria [7.83% (3.43–17.9), median (P25-P75)], and this phenomenon was not dependent on the healthy status in each individual study (Fig. S5). These data indicate that variation across studies may be led by the different detection efficiencies of certain microbial species due to the DNA extraction and sample storage methods.

To investigate the contribution of the sample operating approach to the detection efficiency of certain bacterial lineages, we next calculated the bacterial richness within the dominant bacterial phyla across studies. The Chao1 richness showed large variations across individual studies for these phyla (*P* < 2e−16, KW test, Fig. 2c). The species richness in the gram-positive phyla Firmicutes and Actinobacteria was positively correlated with the overall richness across all the studies (*P* < 2e−16, Spearman’s correlation, Fig. S6). Similar to the alpha diversity observed in the overall bacterial richness, the Promega Kit-extracted samples also exhibited significantly higher certain lineages’ richness within phyla Firmicutes and Actinobacteria compared to the Qiagen Kit (*P* < 2e−16, Mann-Whitney test, Fig. 2c). For samples extracted with the same DNA extraction method, significantly lower richness within Firmicutes and Actinobacteria phyla was observed in preservative than fresh-frozen samples [Qiagen, S1 (fresh frozen) vs S2 (preservative); Promega S3, 5–7 (fresh frozen) vs S4 and 8 (preservative), *P* < 0.05, Mann-Whitney test with Bonferroni correction]. At the species level, multiple species from genera *Actinomyces*, *Streptococcus,* and *Lactococcus* were underdetected with Qiagen, while some species assigned to genera *Bacteroides* and *Butyricimonas* were underdetected with Promega (Fig. 2c and d; Fig. S7). In addition, the recovery rate of *Fusobacterium* spp. was significantly lower in Promega-extracted samples compared with Qiagen-extracted samples in two studies involving CRC patients (study S1 vs S6, *P* < 0.05, KW test, Fig. S8). These results further confirmed that the differences in microbial diversity across studies were mainly contributed by the different DNA extraction efficiencies of certain bacterial species. Inconsistent sample operating approach may cause significant confounders for the microbiome comparison across studies.

### The DNA extraction methods affected microbiome composition in test samples and mock communities

**Fig 3:**
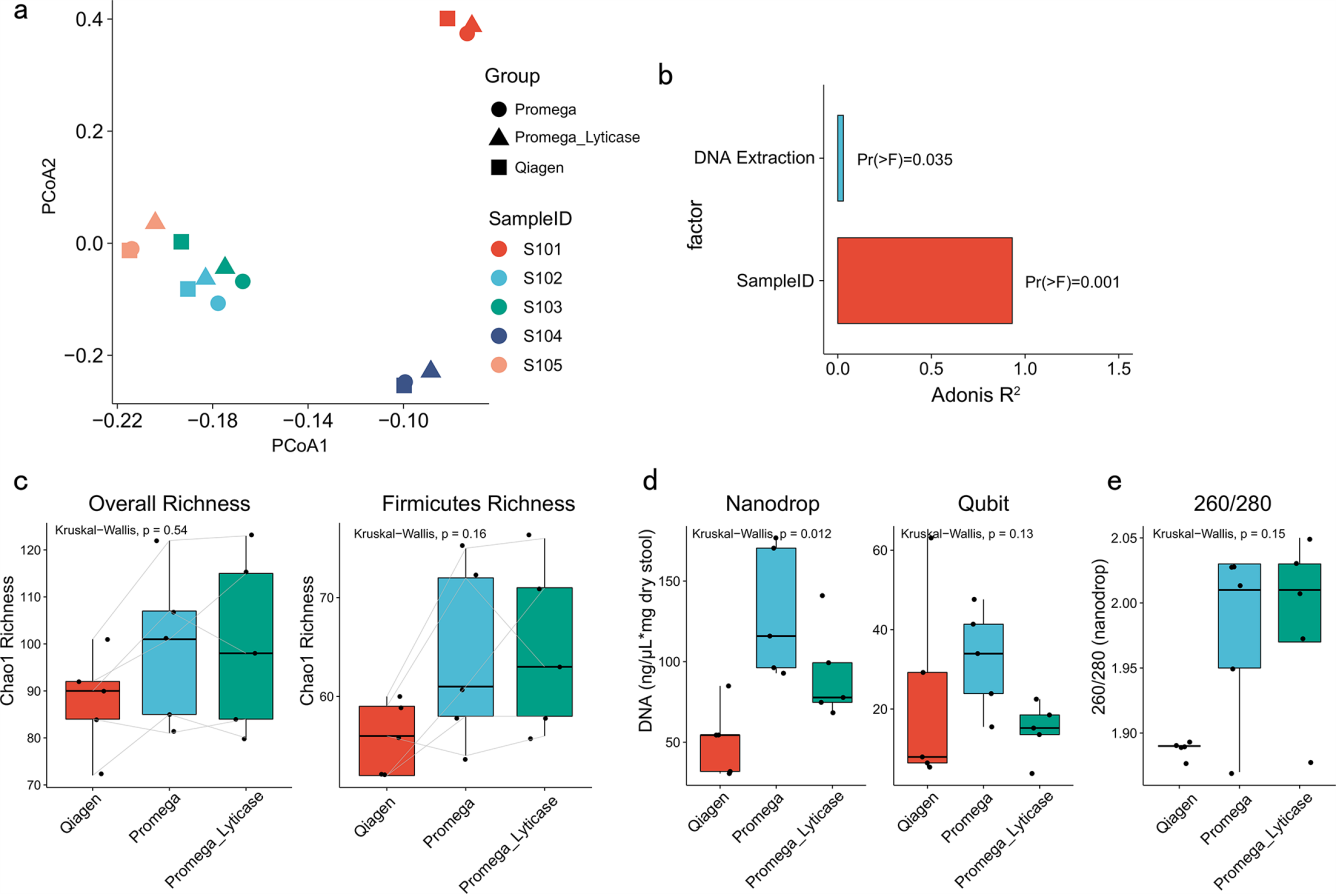
The microbiome profile of test samples extracted using different extraction methods. Five fecal samples from five healthy subjects were extracted with the Qiagen PowerSoil Kit, Promega Maxwell PureFood Kit, and Promega Maxwell PureFood Kit with lyticase pretreatment. (**a**) PCoA analysis based on the weighted UniFrac distance of test fecal samples. (**b**) Bar plot illustrating the factors found to be significantly associated with gut microbial variations. (**c**) Overall richness and richness within the Firmicutes phylum of test fecal samples. (**d,e**) DNA quantity and quality of test samples.

Based on the observation that the DNA extraction methods might be the major contributions in the microbial profiles across different studies, we next evaluated the variation in community composition associated with DNA extraction methods by comparing fecal DNA samples from five healthy individuals (test samples) extracted using the Qiagen PowerSoil Kit or the Promega PureFood Kit, with or without lyticase pretreatment. A mock community with predefined composition was also included in each extraction method. We found that the variation between samples outweighed that attributable to the DNA extraction methods in the test microbial community (PERMANOVA test, *R*^2^ = 0.93, *P* < 0.001 vs *R*^2^ = 0.03, *P* = 0.035, Fig. 3a and b). These results further highlighted the crucial value of controlling DNA extraction methods in microbial data interpretation.

To further confirm the observations in the real data set, we next calculated the alpha diversity overall, or within each bacterial phylum, respectively, in the test fecal microbial community. Similar to the previous findings observed in the real data sets, we observed an increase of both the overall richness and Firmicutes richness in samples extracted with Promega Kit with or without lyticase pretreatment (Fig. 3c). In addition, the Firmicutes richness was positively correlated with the overall richness (*P* < 2e−16, Spearman’s correlation, Fig. S9). The DNA extraction yield of the Promega Kit was significantly higher compared to that of the Qiagen Kit (Fig. 3d). Even so, the Qiagen Kit generated an amount of DNA with better quality compared to the Promega Kit (Fig. 3e).

**Fig 4:**
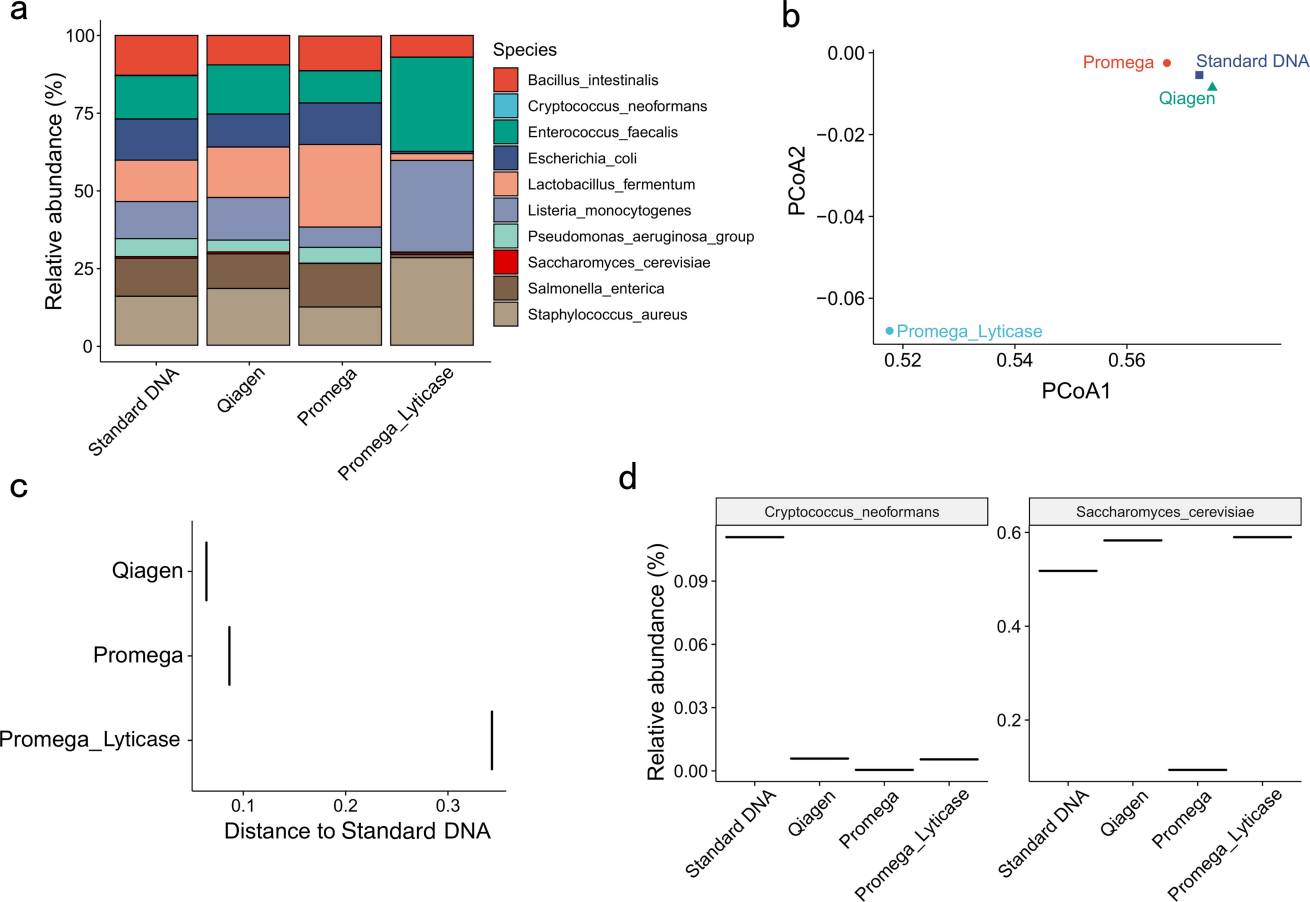
The microbiome profile of the mock community extracted using different extraction methods. The standardized mock community sequencing control (ZymoBIOMICS Microbial Community) was extracted with Qiagen PowerSoil Kit, Promega Maxwell PureFood Kit, and Promega Maxwell PureFood Kit with lyticase pre-treatment. (**a**) Bar plot depicting the composition of mock community samples extracted using different extraction methods. (**b**) PCoA analysis based on the weighted UniFrac distance of mock community samples. (**c**) Weighted UniFrac distance of mock community samples to the standard DNA. Smaller values indicate more resemblance to the standard DNA. (d) Relative abundance of fungal species in mock community samples.

When looking at the microbiome profile of the mock community, samples extracted with the Qiagen or Promega Kit resembled DNA similar to the standard DNA samples, and the theoretical composition was in good agreement with the manufacturer (Qiagen: 0.064 and Promega: 0.086, Fig. 4a through c). In contrast, the lyticase pretreatment resulted in a significant shift in microbiome composition (Fig. 4a through c). Notably, the lyticase pretreatment, which was applied to increase the efficacy of fungal DNA extraction, did increase the abundance of fungal DNA in samples extracted with the Promega Kit but only reached similar levels as samples extracted with the Qiagen Kit (Fig. 4d). These results indicated that the DNA extraction methods influence significantly on microbial profiles by affecting DNA extraction yield, quality, microbial alpha diversity, beta diversity, and efficiency of certain microbial lineages.

## DISCUSSION

Meta-analysis of multiple microbiome cohorts is essential to generate generalizable findings. However, significant biases are often introduced by multiple and inconsistent experimental steps across human microbiome studies (8). It is often suggested that applying standardized protocols addresses issues of between-study bias (26). Here, we used a real-life example of a collated metagenomic data set including 2,722 samples and demonstrated that biases exist among samples processed in the same laboratory. To our knowledge, our study is the first to compare multiple variables, including various host factors (BMI, age, etc.) and sample preparation protocols, in such a large cohort using metagenomic sequencing method of stool samples for microbiome assessment. Our findings suggest that using consistent protocols on similar sample types is critical to compare microbiome across different studies.

Among host factors and sample preparation protocols, DNA extraction methods had the largest effect on gut microbiome composition, aligning with a previous assessment of variation in microbial community amplicon sequencing (27). Although we applied analysis to adjust for batch effects, compositional differences across studies were still statistically significant. In addition, we observed a small but still significant difference in overall microbiome composition in age/BMI-matched healthy subjects recruited for individual studies. During the evaluation of the DNA extraction method on test samples, we also detected a significant difference in overall microbiome composition due to the DNA extraction protocols, albeit the effect size was much smaller than that in the real data sets. In addition to the DNA extraction methods, fresh-frozen samples appear to be a better way to recover both the overall richness and specific bacterial lineages richness. This indicates that other underlying factors besides the DNA extraction kit, such as storage conditions and duration, may also contribute to inter-study variations in microbiome composition. One implication is that healthy controls in case-control microbiome studies must be recruited in parallel with case samples for valid comparisons. If pre-existing control samples are included, they should be checked for consistency with later recruited control samples to ensure that batch differences do not confound biological signals (28). While the preference of these methods may not mask the identification of differential microbes in case-control study settings, combining data sets generated using different DNA extraction methods should be handled with extra caution.

The inter-study variations generally stem from multiple areas, including the inability to quantify extraction efficiencies among different microbes (9). Our data suggest that the inter-study variations may primarily be caused by reduced detection of certain bacterial groups due to the DNA extraction procedure. The Promega Kit has been employed as the primary protocol for DNA extraction in our laboratory since 2018. The Promega Kit was reported to produce higher concentrations with a lower fecal sample input weight compared to the Qiagen protocol (6, 29). It indeed increased the success rate of library preparation for us in the past (data not shown) and increased the yield of extracted DNA from the test samples in this study. As expected, the dominant phylum observed with Promega was Firmicutes (gram positive, median relative abundance of 71.2%), as opposed to the dominant phylum observed with Qiagen, which was Bacteroidetes (gram negative, median relative abundance of 68.3%). The DNA extracted with the Promega Kit also had higher overall richness and Firmicutes richness in both real data sets and test samples, compared with DNA extracted with the Qiagen Kit. Comparative study indicated that the Human Microbiome Project protocol was less effective at extracting DNA from eukaryotes and gram-positive bacterial lineages compared with the MetaHIT project (30). Our findings showed good agreement with what is described in the Promega protocol, which includes mechanical bead beating and enzyme lysis steps crucial for the effective recovery of gram-positive bacteria (6, 10, 31).

In terms of species bacterial lineages, gram-positive species *Actinomyces* spp., *Streptococcus* spp., and *Lactococcus* spp. were underdetected with Qiagen, while gram-negative species *Bacteroides* spp. and *Butyricimonas* spp. were underdetected with Promega. Consistently, for the mock microbial community, the relative abundance of the gram-positive *Lactobacillus* also increased in the samples extracted with the Promega Kit. In addition to the dominant bacterial phyla, the recovery rate of *Fusobacterium* spp. was significantly lower in Promega-extracted samples compared with Qiagen-extracted samples. Our results demonstrated that different DNA extraction protocols should be considered when designing experiments if there are specific gut taxa of interest (i.e., *Fusobacterium* in CRC microbiome research).

The need for standardized methodologies is frequently emphasized in the microbiome field to make it easier and more robust to compare results from different studies (6). However, there are no perfect methodologies, and all are biased in some way (32, 33). Our data provide some guidance on how to decide which protocol researchers should use when designing experiments, as the “best” method fundamentally depends on the underlying structure of the microbial community, and this can vary hugely between individuals (33). While our study provides valuable insights into the importance of standardized methodologies in microbiome research, it is important to acknowledge several limitations. First, the majority of samples in this study utilized the Promega DNA Extraction Kit, which may not represent the full spectrum of available methodologies. Second, regarding the comparisons between protocols, we tested three protocols: Promega, Promega with lyticase, and Qiagen, to mimic the reality of our data sets, which predominantly used these methods. A more comprehensive controlled laboratory study, including a broader range of DNA extraction kits and storage methods, would provide a stronger argument. Additionally, while we suggest that sample operating procedures should be consistent across different cohorts to ensure comparability, practical constraints often make this difficult. In such cases, we recommend benchmarking protocols across studies to identify consistent patterns of bias. Therefore, detailed methodology should be properly recorded following the STORMS checklist (Strengthening the Organization and Reporting of Microbiome Studies) (34). This would allow researchers with similar research interests to use consistent protocols in the same disease settings and is especially important when trying to define a “healthy microbiome” or reference ranges for microbial lineages in certain populations.

Overall, multiple steps involved in the sample operating approach are crucial for getting comparable microbiome data across studies without bias. Thus, it is highly recommended that all sample operating procedures in different cohorts should be kept consistently if they were meant to form a large cohort for microbiome work. If this is impossible, protocols should be benchmarked across studies to identify whether consistent patterns of bias can be found. This will give researchers adjustable information when comparing results between studies. Additionally, batch effects should be considered for cohorts with large sample size or longitudinal cohorts. Finally, all biological conclusions generated from sequence-based microbiome data sets should be validated through other molecular techniques to ensure that the results and underlying mechanisms are robust regardless of biases due to inconsistent sample processing methods during microbiome sequencing experiments.

## Data Availability

Raw sequencing data are available from the National Center for Biotechnology Information Bio-Projects (accession number PRJNA648796, PRJNA648797, PRJNA557323, PRJNA686821, PRJNA943687, PRJNA650244).

## SUPPLEMENTAL MATERIAL

Supplemental figures spectrum.01516-24-s0001.pdf Figures S1 to S9.

Supplemental legends spectrum.01516-24-s0002.docx Legends for Fig. S1 to S9.

ASM does not own the copyrights to Supplemental Material that may be linked to, or accessed through, an article. The authors have granted ASM a non-exclusive, world-wide license to publish the Supplemental Material files. Please contact the corresponding author directly for reuse.
