# Supplementary figures and images for: Variation in the metagenomic analysis of fecal microbiome composition calls for a standardized operating approach
Source: Microbiol Spectr. 2024 Oct 30;12(12):e01516-24. doi: 10.1128/spectrum.01516-24 (PMC11619352; doi:10.1128/spectrum.01516-24)

Figure S1

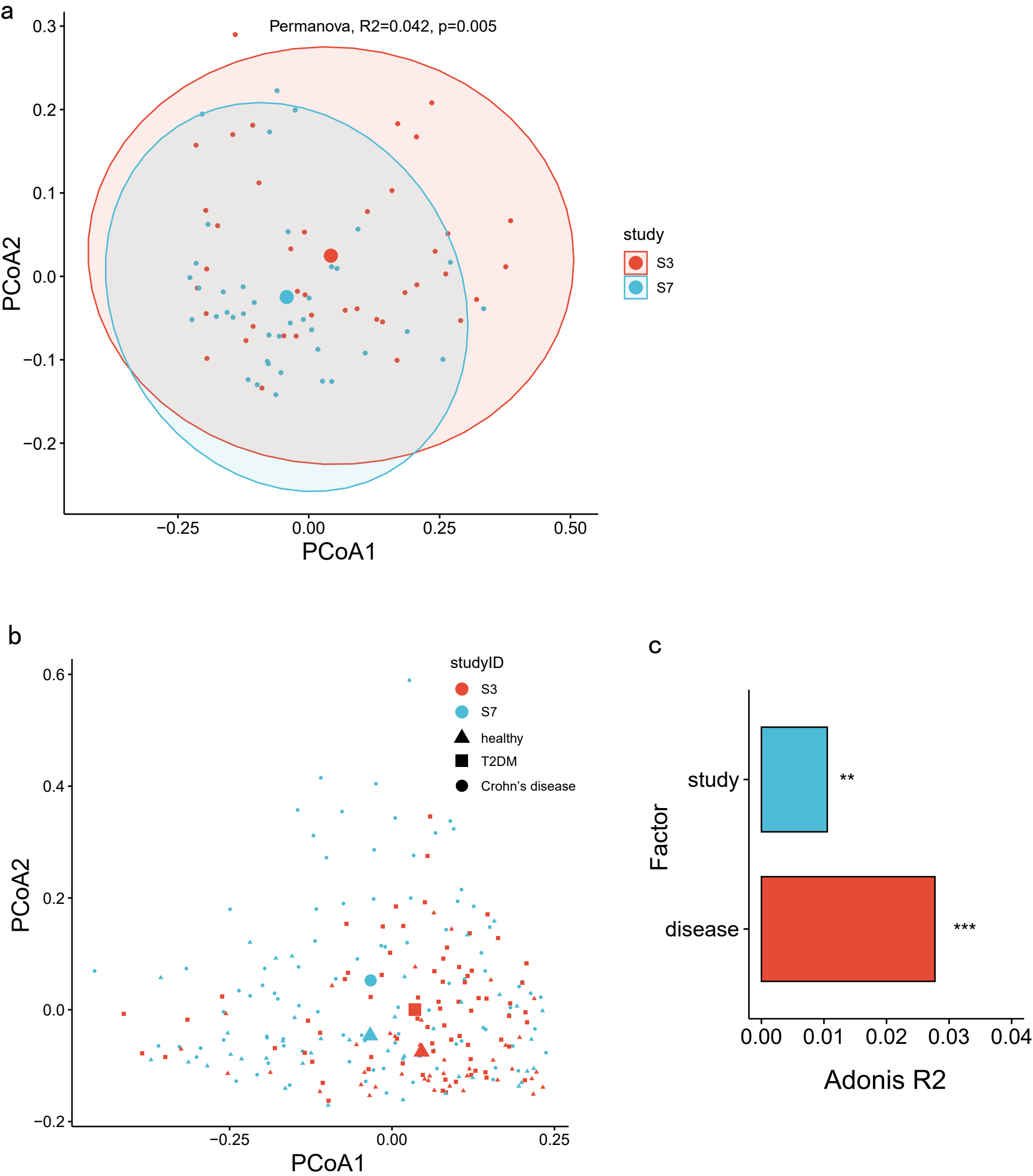

# Figure S2

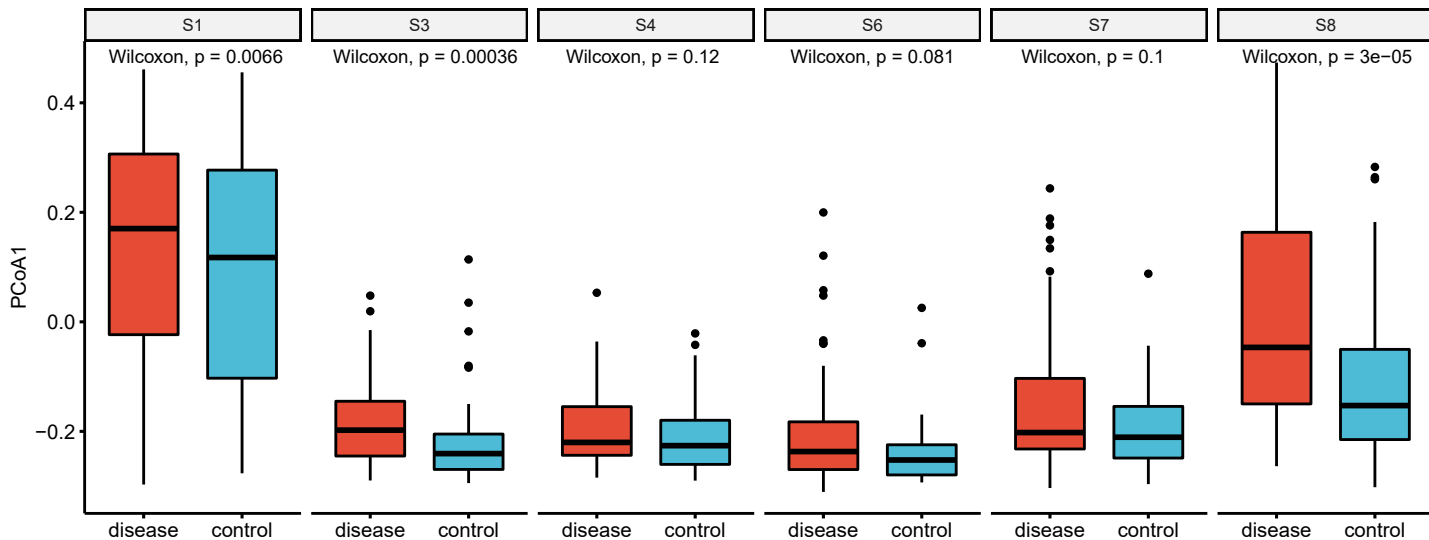

# Figure S3

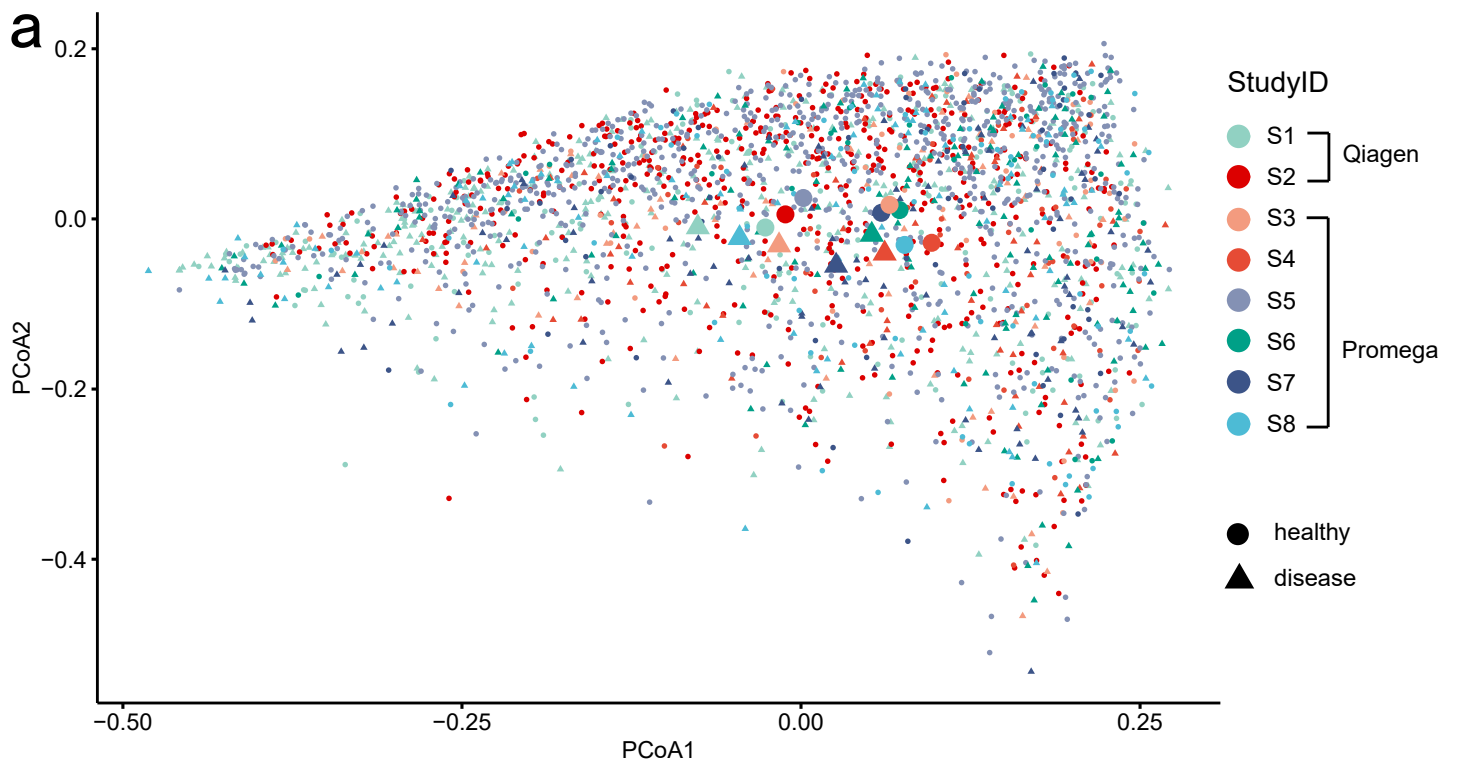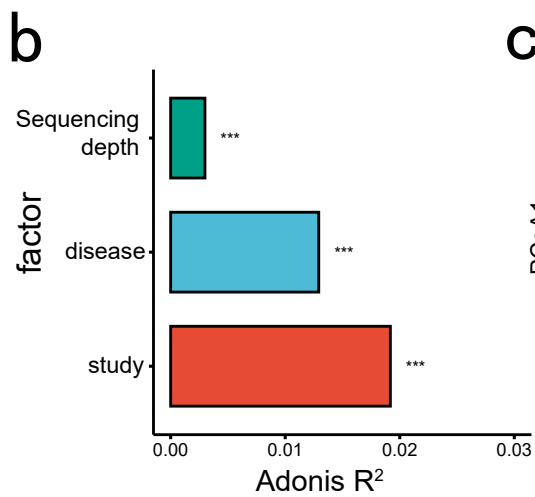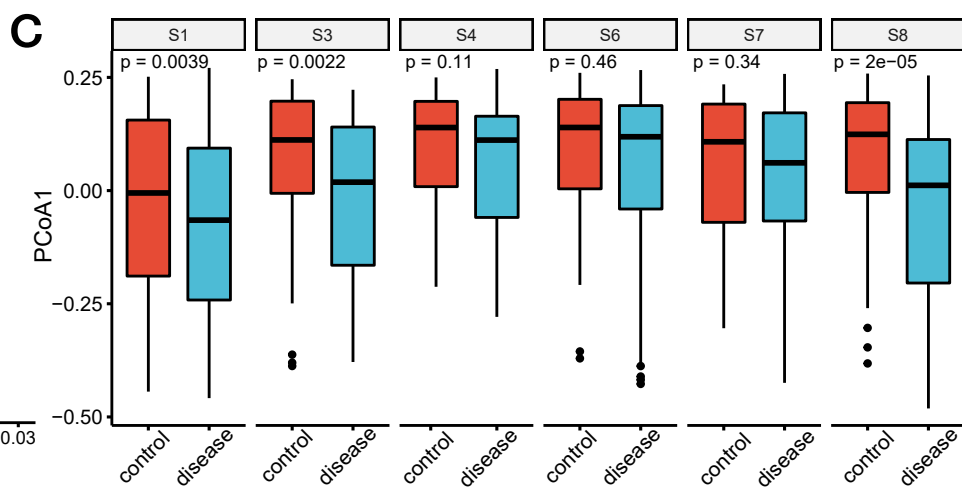

Figure S4

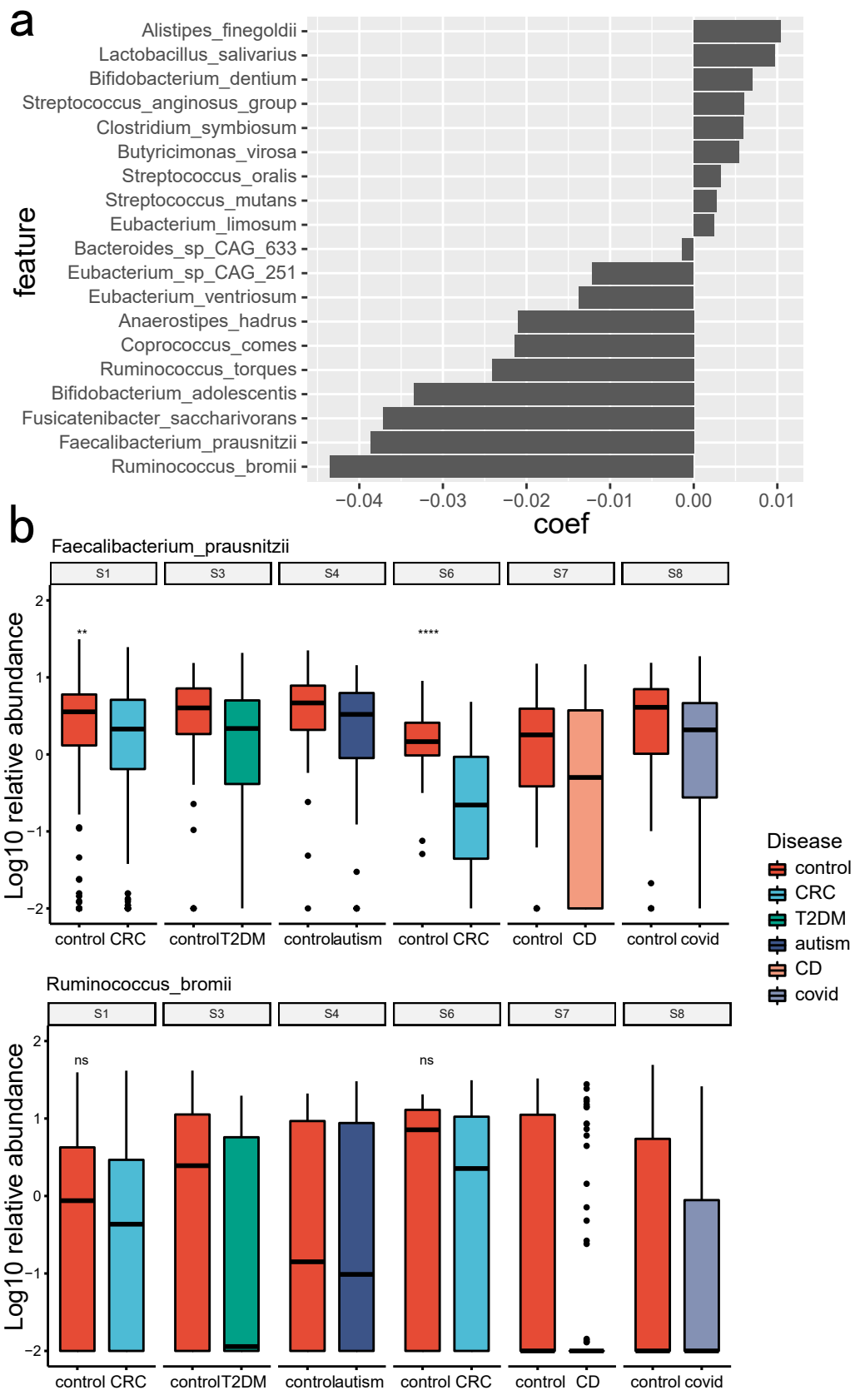

Figure S5

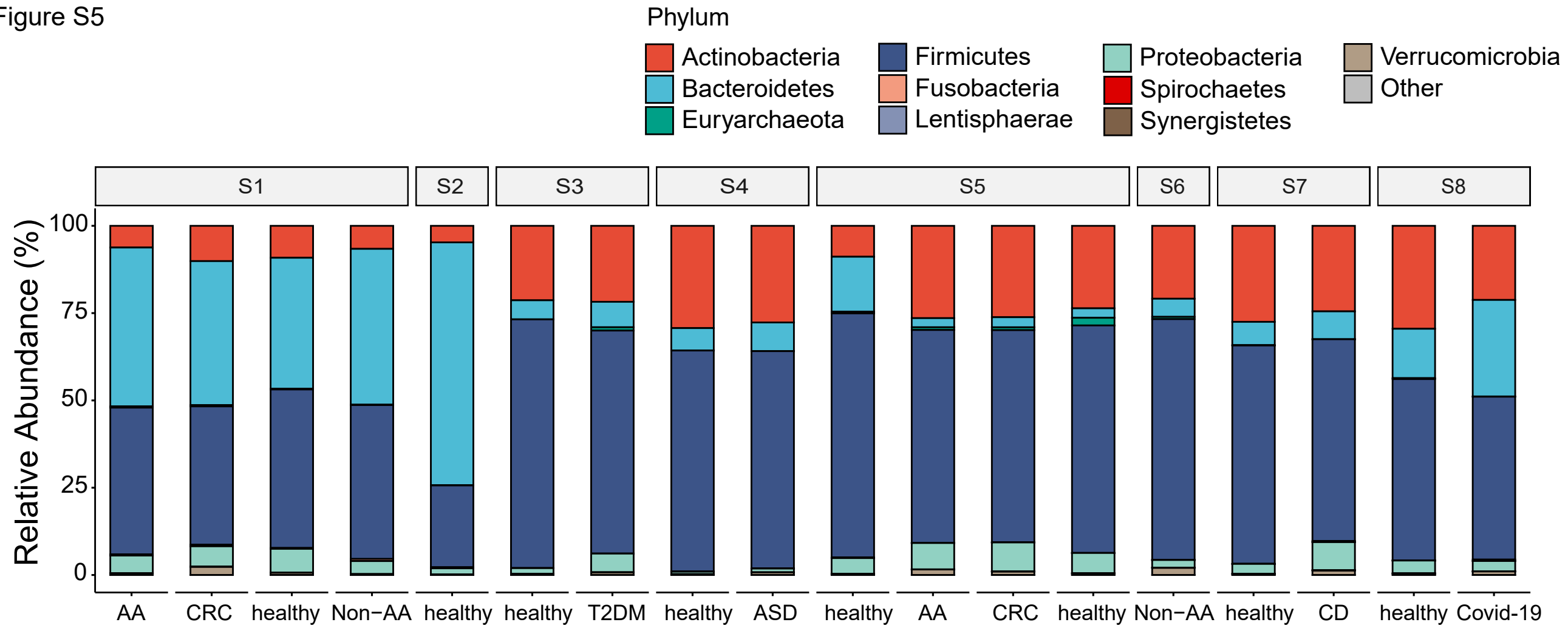

# Figure S6

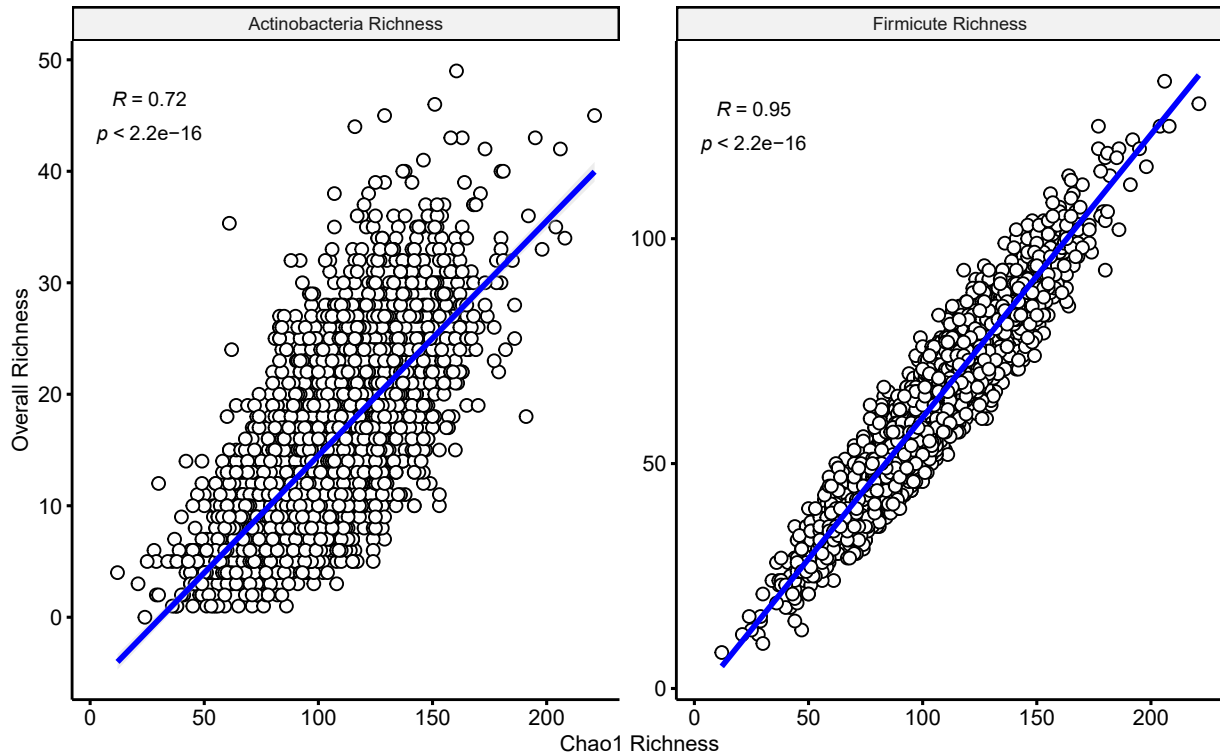

## Figure S7

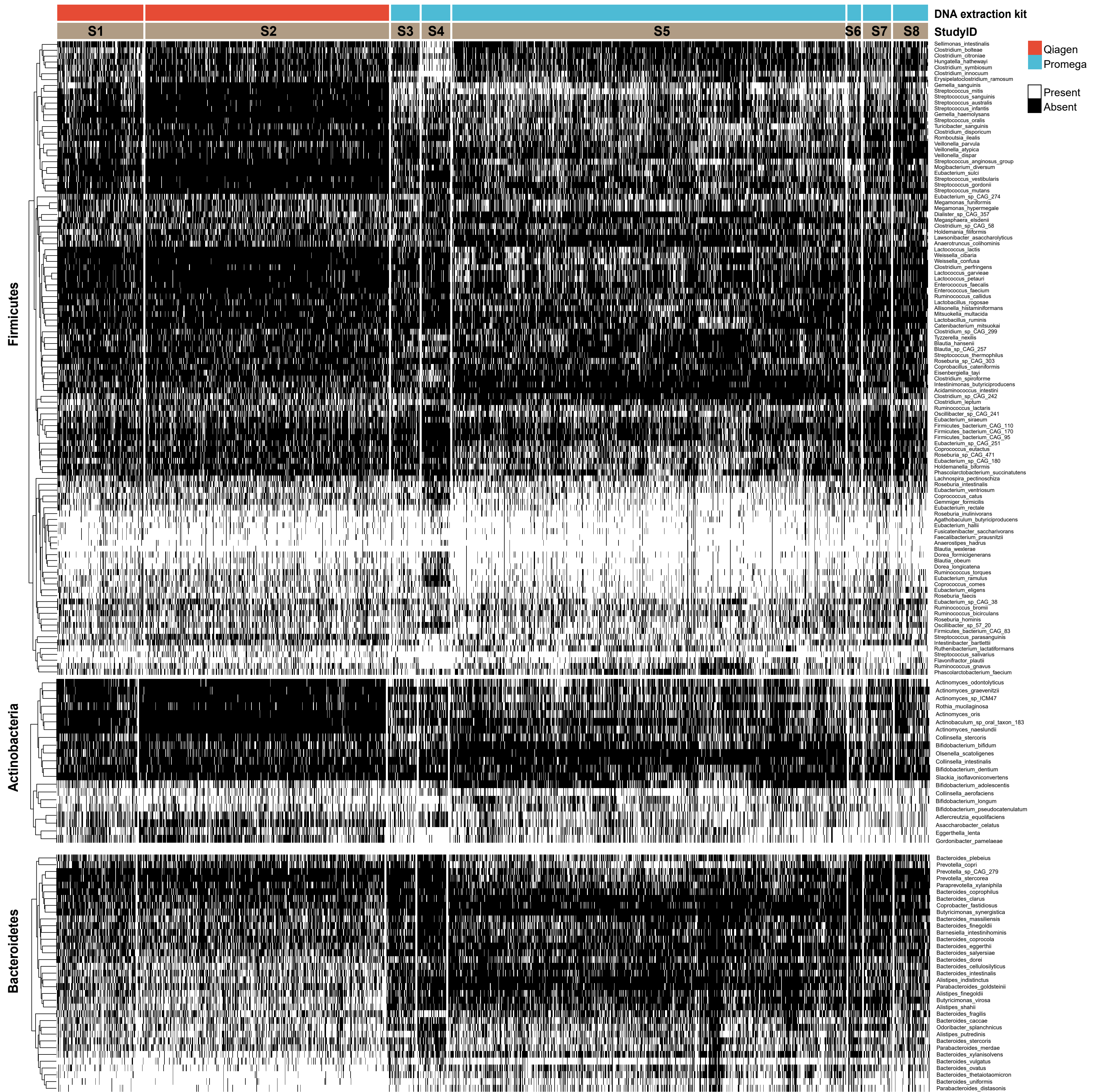

# Figure S8

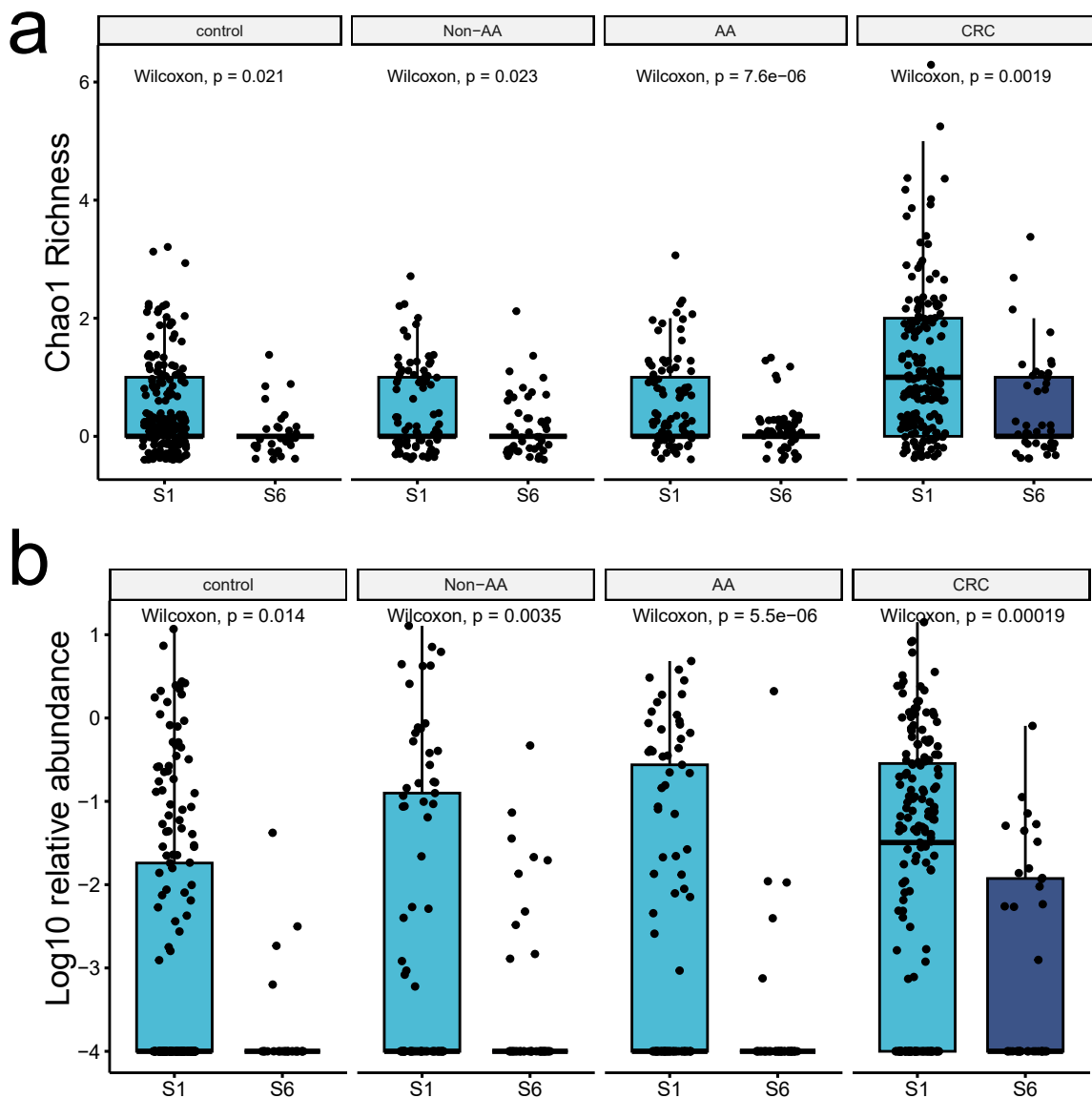

Figure S9

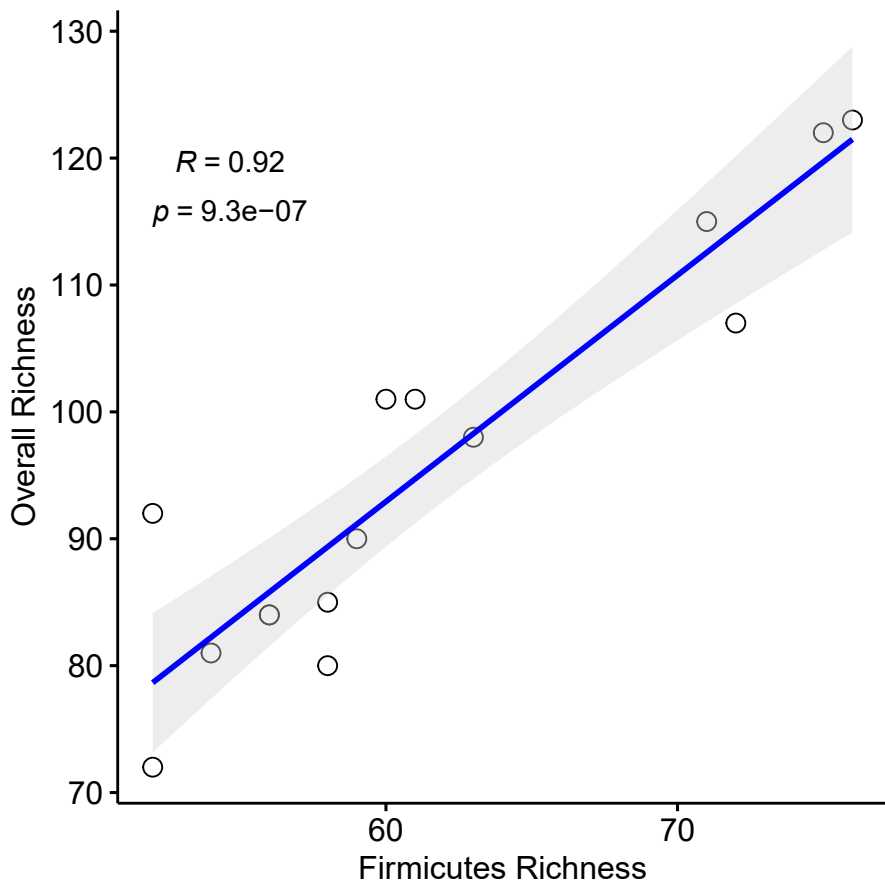

Supplement: Supplemental figures — Figures S1 to S9. [file spectrum.01516-24-s0001.pdf]
