# Supplementary material for: Variation in the metagenomic analysis of fecal microbiome composition calls for a standardized operating approach
Source: Microbiol Spectr. 2024 Oct 30;12(12):e01516-24. doi: 10.1128/spectrum.01516-24 (PMC11619352; doi:10.1128/spectrum.01516-24)
Supplement: Supplemental legends — Legends for Fig. S1 to S9. [file spectrum.01516-24-s0002.docx]

**Supplementary Figure Legend**

**Supplementary Figure 1**

a. Principal coordinates plot based on weighted-unifrac distance showing among matched samples from control subjects using Promega kit. Samples were matched by propensity score. b. Principal coordinates plot based on weighted-unifrac distance showing among matched samples from both control subjects and diseased subjects using Promega kit. c. Bar plot illustrating the Variance explained (R2) by each factor associated with gut microbial variations among matched samples. R2 and statistical significance were calculated by PERMANOVA (Adonis2). FDR was controlled at 5%. Factors were ranked by R2. All samples were fresh-frozen. DNA were extracted with Promega kit without lyticase pretreatment. N=40 pairs.

**Supplementary Figure 2**

Barplot depicting the distribution of diseased subjects compared with healthy controls in their respective study along the PCoA1 axis. Statistical significance was calculated by Wilcoxon rank sum test.

**Supplementary Figure 3**

a. Principal coordinates plot based on weighted-unifrac distance showing variation among samples from individual studies after batch effect adjustment. b. Bar plot illustrating the factors found to be significantly associated with gut microbial variations. c. Barplot depicting the distribution of diseased subjects compared with healthy controls in their respective study along the PCoA1 axis.

**Supplementary Figure 4**

a. Differentially abundant species between healthy and diseased subjects by lm_meta function in MMUPHin package (FDR adjusted p<0.05). b-c. Boxplot depicting the relative abundance of top two species, Faecalibacterium prausnitzii (b) and Ruminococcus bromii (c) that were shown to associate with disease status. Statistical significance was calculated by Wilcoxon rank sum test.

**Supplementary Figure 5**

Bar plot depicting the relative abundance at phylum level across included studies.

**Supplementary Figure 6**

Scatter plot depicting the correlation of the richness within phyla Firmicutes and Actinobacteria with the overall richness. Statistical significance was calculated by Spearman’s correlation.

**Supplementary Figure 7**

Heatmap depicting the presence and absence of bacterial species within major bacterial phyla across included studies. Black cells represent species that were undetected, whereas white cells represent species that were detected in a given sample.

**Supplementary Figure 8**

a. Boxplot depicting the richness of *Fusobacterium spp.* in Promega-extracted samples and Qiagen-extracted samples in studies involving CRC patients. b. Boxplot depicting the abundance of *Fusobacterium spp.* in Promega-extracted samples and Qiagen-extracted samples in studies involving CRC patients.

**Supplementary Figure 9**

Spearman’s correlation of the richness within phyla Firmicutes with the overall richness in the test fecal microbial community.
